# Supplementary material for: WordCommentsAnalyzer: A windows software tool for qualitative research
Source: F1000Res. 2018 Sep 4;7:536. Originally published 2018 May 3. [Version 2] doi: 10.12688/f1000research.14819.2 (PMC6137411; doi:10.12688/f1000research.14819.2)
Supplement: Supplementary file 3 [file f1000research-7-17614-s0002.tgz › b2a79496-4ada-47c4-9682-a99ce867836d.zip!/Eight Iranian Health Journals/Journal of Knowledge and Health_29.docx]

© Journal of Knowledge and Health. This work is licensed under CC BY 4.0 (https://creativecommons.org/licenses/by/4.0/).

Volume & Issue: دوره 6، شماره 1: 1390

Title: بررسی تأثیرعصاره گیاه رازیانه بر علايم سندروم پیش از قاعدگی

http://knh.shmu.ac.ir/index.php/site/article/view/87

مقدمه: سندروم پیش از قاعدگی (PMS) را اغلب زنان در سن باروری تجربه می‌کنند. این سندروم یک رخداد دوره‌ای بوده و ترکیبی از تغییرات فیزیکی، روانی و رفتاری را شامل می­شود و با ارتباطات خانوادگی و فعالیت‌های اجتماعی تداخل می‌کند. در این راستا روش‌های درمانی متفاوتی مطرح شده و یکی از این روش‌های درمانی استفاده از گیاهان دارویی به­صورت سنتی می‌باشد. این مطالعه با هدف تعیین تأثیرعصاره رازیانه بر علايم سندروم پیش از قاعدگی در دانشجویان دانشگاه علوم پزشکی شهرکرد انجام شد. مواد و روش­ها: در این کارآزمايی بالینی یک سو کور60 نفر از دانشجویان دارای علايم PMS به‌صورت تصادفی به دو گروه 30 نفره دریافت‌کننده رازیانه و دارونما اختصاص داده شدند. ابزار جمع‌آوری اطلاعات پرسش­نامه ثبت روزانه شدت مشكلات (Daily Record of Severity of Problems) بود و شدت این سندروم در مدت 2 ماه قبل از درمان تعیین و با شرایط بعد از درمان مقایسه شد. جهت تحليل اطلاعات از نرم‌افزار SPSS استفاده و ميزان 05/0>P از نظر آماری معنادار در نظر گرفته شد. نتايج: دو گروه از نظر میانگین امتیازات علايم PMS قبل از مداخله با یکدیگر تفاوت معنادار آماری نداشتند ]‌‌‌(4/33±4/101 در گروه رازیانه در مقابل 5/19±0/104 در گروه دارونما،61/0=P )[، اما تفاوت بین دو گروه پس از مداخله از لحاظ آماری معنادار بود،]4/29±4/64 در گروه رازیانه در مقابل 1/28±1/79، در گروه دارونما 01/0=[P. نتیجه‌گیری: عصاره رازیانه احتمالاً بر کاهش علايم PMS مؤثر است. استفاده از آن در درمان این سندروم پیشنهاد می‌گردد.

Title: سیمای سوء تغذیه در کودکان شهرستان شاهرود

http://knh.shmu.ac.ir/index.php/site/article/view/88

مقدمه: با وجود روند کاهشی سوء تغذیه در کودکان ایران، این پدیده هنوز یکی از مشکلات جدی بهداشتی در مناطق مختلف کشور می‌باشد. اگرچه می‌توان بر اساس مطالعات ملی برآوردی از وضعیت سوء تغذیه در شهرستان شاهرود به­دست آورد اما اطلاعی از وضعیت مناطق مختلف شهرستان از این نظر در دسترس نیست. هدف از انجام این مطالعه بررسی شیوع لاغری، کم وزنی و کوتاه قدی در کودکان زیر 6 سال شهرستان شاهرود می­باشد. مواد و روش‌ها: به روش نمونه‌گیری خوشه‌ای ‌1395 کودک زیر 6 سال‌، در 13 منطقه جغرافیایی مختلف شهرستان به‌صورت تصادفی انتخاب و اندازه‌گیری قد و وزن در مورد آن‌ها انجام و از طریق مصاحبه با مادران آن‌ها، سایر داده‌های مورد نیاز به‌دست آمد. شاخص‌های سوء تغذیه با استفاده از نرم‌افزارAnthro و بر پایه جامعه مرجع WHO در سال 2007 محاسبه شد. نتایج: شیوع لاغری، کم وزنی و کوتاه قدی یعنی درصد کودکانی که Z-score آن‌ها در مورد این شاخص‌ها ‌کم‌تر از 2- بود، به‌ترتیب 7/4%، 7/5% و 3/10% گزارش شد. شاخص نمايه توده بدني براي سن نشان داد که 4% از کودکان دارای Z-score بیشتر از 2 و دارای اضافه وزن هستند. توزیع این شاخص‌ها در نقاط مختلف شهرستان بسیار متفاوت بود. کوتاه قدی در منطقه كالپوش و بيارجمند از شهرستان شاهرود در 22% کودکان ديده شد. نتیجه‌گیری: شیوع سوءتغذیه در شهرستان شاهرود نسبت به بقیه مناطق استان سمنان بیش‌تر و نابرابری بزرگی در توزیع این حالت در مناطق مختلف وجود دارد. به‌کارگیری مداخلات جامع و محلی در این زمینه ضروری به نظر می­رسد.

Title: بررسي ارتباط بين شاخص توده بدني و عوامل دموگرافيك در زنان

http://knh.shmu.ac.ir/index.php/site/article/view/89

مقدمه: چاقي يک وضعيت پيچيده با ابعاد مهم رواني و اجتماعي است که در همه سنين و در همه گروه‌هاي اقتصادي و اجتماعي ديده مي‌شود. چاقي يک عامل خطر تغييرپذير براي بسياري از بيماري‌هاي مزمن مي‌باشد. براي بررسي اضافه وزن و چاقي استفاده از نمايه توده بدني (BMI) روشي معمول و ساده مي‌باشـد. اين مطالعه با هدف تعيين ارتباط بين شاخص توده بدني و عوامل دموگرافيك در زنان مراجعه‌کننده به مراکز بهداشتي- درماني اهواز انجام شد. مواد و روش­ها: مطالعه حاضر يک مطالعه مقطعي مي­باشد. با استفاده از نمونه­گيري تصادفي خوشه­اي 899 نفر از زنان مراجعه‌کننده به مراکز بهداشتي- درماني اهواز در سال 1387 وارد مطالعه شدند. ارتباط متغيرهـاي تحصيلات، وضعيت تأهل، شغل، سن با شـاخص تـوده بـدني (BMI) با روش رگرسيون خطي چندگانه و به کمک نرم­افزار SPSS بررسي شد. نتايج: متغيرهاي سن و وضـعيت تأهل با متوسط BMI ارتباط مستقيمي داشتند که از نظر آماري معنادار بود (0001/0=P). هم‌چنين متغير سال‌هاي تحصيل نيز ارتباط معکوس و معناداري را با BMI نشان داد (005/0=P). ولي ارتباط متغير شغل با BMI در مدل رگسيوني از نظر آماري معنادار نبود (14/0=P). نتيجه‌گيري: افزايش سن، متأهل بودن و کم بودن سطح تحصیلات از عوامل تأثیرگذار بر BMI افراد می‌باشد.

Title: بررسي آگاهي، نگرش و عملكرد تغذيه‌اي دانش‌آموزان دختر مدارس راهنمايي در دو منطقه از ایران

http://knh.shmu.ac.ir/index.php/site/article/view/91

مقدمه: دختران نوجوان مادران آینده هستند و مفاهیم تغذیه‌ای آموخته شده به وسیله آن‌ها به دوران بزرگسالی منتقل می‌شود و نقش مهمی در سلامتی خانواده و کودکان دارد. این مطالعه با هدف ارزيابی آگاهی، نگرش و عملکرد تغذیه­ای دختران دانش‌آموزان دختر مقطع راهنمایی در دو منطقه از ایران انجام شده است. مواد و روش‌ها: در این مطالعه توصیفی تعداد 587 دانش‌آموز دختر در محدوه سنی 11 تا 16 سال در دو شهر شاهرود و ملایر در سال 1388 با روش نمونه‌گیری ساده تصادفی انتخاب و مورد بررسی قرار گرفتند. به‌منظور ارزیابی آگاهی، نگرش و عملکرد تغذيه‌اي افراد از پرسش‌نامه‌ای استفاده شد که حاوی اطلاعات دموگرافیک، آگاهی، نگرش و عملكرد تغذیه‌ای برای نمونه‌ها بود. برآورد میزان آگاهی، نگرش و عملکرد بدین نحو بود که افرادی که امتیازشان بیش‌تر از 75% حداکثر امتیاز بود، به‌عنوان خوب و مطلوب و افرادی که امتيازشان در محدوده‌ی 50-75% حداکثر امتیاز قرار داشتند و افرادی که کم‌تر از 50% حداکثر امتیاز را کسب کرده بودند، به‌عنوان ضعیف در نظر گرفته شدند. نتایج: در مجموع در دو شهر اکثریت افراد دارای آگاهی متوسط (5/59%)، نگرش خوب (2/55%) و عملکرد متوسط (5/55%) بودند. نتیجه‌گیری: نتایج نشان دادکه نوجوانان دختر از آگاهی و عملکرد متوسط برخوردار هستند و با توجه به اینکه عملکرد تغذیه­ای مطابق نگرش آن­ها نیست، ضرورت مداخلات تغذیه­ای مداوم را در نوجوانان آشكار می­سازد.

Title: تأثیر فعالیت جسمانی منظم به‌عنوان یک بخش اساسی اصلاح سبک زندگی بر کاهش عوامل خطر قلبی- عروقی

http://knh.shmu.ac.ir/index.php/site/article/view/92

مقدمه: فعاليت جسماني از ديرهنگام محور اصلي اقدامات مداخله‌اي در كاهش بار تحميلي بیماری‌های قلبی- عروقی بوده است. هدف اين مطالعه‌ بررسي ارتباط بين فعاليت و عدم فعاليت با عوامل خطر بيماري‌هاي قلبي- عروقي در افراد نظامي سالم مي‌باشد. مواد و روش‌ها: شاخص‌هاي تن‌سنجی، تابلوی ليپيد- ليپوپروتئين، فشارخون و قند خون ناشتا در دو گروه فعال (50=n) و غير فعال (50=n) مورد ارزيابي قرار گرفت. نتايج: افراد داراي فعاليت جسماني به‌طور معناداري داراي اندازه‌هاي کم‌تر شاخص‌هاي بدني (به استثناي قد)، سطوح کم‌تر کلسترول تام، ليپوپروتئين با چگالي کم، تري‌گليسيريد، فشارخون سيستوليک و دياستوليک، عامل خطر (نسبت کلسترول تام/ ليپوپروتئين با چگالي بالا)، شاخص آتروژنيک (نسبت ليپوپروتئين با چگالي کم/ ليپوپروتئين با چگالي بالا) و سطوح بيش­تر ليپوپروتئين با چگالي بالا بودند. ضرايب همبستگي ساده نشان داد که شاخص توده‌ بدني و نسبت دور کمر به دور باسن، به­طور معنا‌داري با فشارخون سيستوليک در گروه غير فعال ارتباط مثبت و شاخص توده‌ بدني ارتباط معکوس با ليپوپروتئين با چگالي بالا در هر دو گروه داشت که در گروه غير فعال از ميزان بيش­تري برخوردار بوده و محيط دور کمر ارتباط معکوس با ليپوپروتئين با چگالي بالا در گروه غيرفعال داشت.نتيجه‌گيري: با کاهش عوامل خطر بروز حوادث قلبي- عروقي و پيشگيري قابل توجه از پيشرفت آن، خواص مفيد فعاليت جسماني برجسته مي‌شود و به تأثير آن به­عنوان يک اقدام حمايتي براي برخوردار بودن از سبک زندگي سالم‌تر در جامعه و به­ويژه در ميان پرسنل نظامي مي‌توان تأکيد و اشاره كرد

Title: بررسي سندرم متابوليك در مادران زنان مبتلا به سندرم تخمدان پلي‌كيستيك

http://knh.shmu.ac.ir/index.php/site/article/view/94

مقدمه: تقريباً 40% از خواهران سالم زنان مبتلا به سندرم مبتلا به تخمدان پلی‌کیستیک، داراي فنوتيپ هايپراندورژنميا بوده و داراي اختلالات متابولیکی نيز مي‌باشند. هدف اين مطالعه، بررسي سندرم متابوليك و تستوسترون آزاد در مادران زنان مبتلا به سندرم تخمدان پلي‌كيستيك مراجعه‌كننده به درمانگاه­هاي وابسته به دانشگاه علوم پزشكي شيراز مي‌باشد. مواد و روش‌ها: مطالعه بر روی 34 نفر از مادران زنان مبتلا به سندرم پلی‌کیستیک و 34 نفر از وابستگان زنانی که مبتلا به سندرم پلی‌کیستیک نبودند، صورت گرفته است. متغیرهای فشارخون، قد و وزن، سطح سرمی انسولین، قند خون، تستوسترون و لیپوپروتئین‌ها در دو گروه مورد بررسی قرار گرفتند. سندرم متابوليك طبق شاخص‌هاي ATPIII(Adult Treatment Panel Ш) و (International Diabetes Federation)IDF ، مقاومت به انسولین طبق نمايه­هاي HOMA (Homeostasis Model Assessment Index)، QUICKI (Quantitative Insulin Sensitivity Check Index) و انسولين ناشتا، kg/m2 30≤BMI، مورد ارزیابی قرار گرفت. نتايج: فراواني نسبي سندرم متابوليك طبق شاخص ATPIII و  IDFدر مادران زنان مبتلا به سندرم پلی‌کیستیک با فراواني نسبي این سندرم در وابستگان زنان سالم تفاوت آماری معناداری نداشت (05/0P>). ميانگين قند خون ناشتا، فشارخون، تستوسترون خون، كلسترول تام، LDL خون، تري‌گليسريد در مادران زنان مبتلا به سندرم پلی‌کیستیک به‌طور معناداري بالاتر از وابستگان زنان سالم بود (05/0P<). چاقي مركزي در دو گروه تفاوت آماری معناداری با یکدیگر نداشت (05/0<P). نتیجه‌گیری: وابستگان درجه یک زنان مبتلا به سندرم تخمدان پلی‌کیستیک به‌خصوص مادران آن‌ها در معرض خطر ابتلاء به افزایش قند خون و اختلالات چربی‌های خون می‌باشند.

Title: بررسی تأثیر گروه درمانی شناختی برکاهش افسردگی دانش‌آموزان

http://knh.shmu.ac.ir/index.php/site/article/view/96

مقدمه: متناسب با سبب شناسي افسردگي در نوجوانان شيوه­هاي درماني مختلفي شناخته شده است که یکی از آن‌ها شناخت درمانی است. هدف از مطالعه حاضر بررسی تأثیر شناخت درمانی گروهی بر کاهش افسردگی دانش­آموزان مقطع متوسطه است. مواد و روش‌ها: در این پژوهش از بین کلیه دانش‌آموزان پسر مقطع متوسطه یک مرکز شبانه‌روزی در منطقه طارم استان قزوین، 16 دانش‌آموز با روش تصادفی ساده انتخاب و به‌طور تصادفی به دو گروه مداخله و شاهد اختصاص داده شدند. ابزار پژوهش پرسش‌نامه افسردگی بک بود. اعضای گروه مداخله در 8 جلسه 90 دقیقه‌ای شناخت درمانی شرکت کردند و گروه شاهد هیچ درمانی دریافت نکردند. برای مقايسه ميانگين نمرات دو گروه از آزمون t مستقل استفاده شده است. نتایج: نتایج نشان داد که، بین میانگین نمرات پیش آزمون و پس آزمون گروه مداخله و شاهد تفاوت معناداری وجود دارد. بدین معنی که شناخت درمانی گروهی موجب کاهش ميانگين نمره افسردگی دانش‌آموزان در گروه مداخله نسبت به گروه شاهد شده است (1/2- در مقابل 25/0-). نتیجه‌گیری: يافته­هاي پژوهش حاضر مشخص مي‌كند كه شناخت درمانی گروهی می‌تواند علايم افسردگی دانش‌آموزان را كاهش دهد. بنابراين از اين يافته مي‌توان در راستاي برنامه‌ريزي درماني یا استفاده از چهارچوب نظریه شناختی در کاهش و یا جلوگیری از افزایش افسردگی دانش‌آموزان استفاده کرد.

Title: مفهوم شأن بیمار و عوامل مرتبط با آن: یک مطالعه متاسنتز کیفی

http://knh.shmu.ac.ir/index.php/site/article/view/97

مقدمه: شأن در کدهای اخلاقی به‌عنوان یک وظیفه و در چارچوب حقوق انسانی به‌عنوان یک حق تلقی می‌گردد. مطالعه متاسنتز حاضر با هدف بررسی مفهوم شأن بیمار و عوامل مرتبط با آن در طی سال‌های 2010-2000 صورت گرفته است. مواد و روش‌ها: در مطالعه متاسنتز حاضر که به‌صورت سیستماتیک صورت پذیرفت، مقالات کیفی منتشر شده پیرامون عوامل مرتبط با مفهوم شأن بیمار در طی سال‌های 2010-2000 میلادی در بانك‌های اطلاعاتی Medline، Elsevier و Ovid انتخاب گردید. براي تسهيل جستجو، ابتدا در بانك اطلاعاتی Medline، با استفاده از سیستم MeSH، عبارت "Patient dignity" استفاده گرديد. در مرحله بعد، با مرور متن کامل مقالات، مقالاتی كه دقیقاً مفهوم شأن بیمار یا عوامل مرتبط با آن را مورد بررسی قرار می‌دادند و مفهوم شأن در عنوان مقالات ذکر شده بود و هم‌چنین به زبان انگلیسی منتشر شده بودند، براي بررسي انتخاب و با يكديگر مورد مقایسه قرار گرفتند. نتایج: تعداد 620 مطالعه کیفی در ارتباط با مفهوم شأن بیمار یافت شد که از این تعداد، 64 مطالعه مستقیماً در ارتباط با موضوع پژوهش و شامل: 21 مطالعه مربوط به مدلاین، 3 مطالعه مربوط به Elsevier و 40 مطالعه از Ovid بود. در کل از میان 64 مقاله یافت شده در طی سال‌های 2010-2000، تعداد 16 مقاله تمام متن انتخاب گردید و با رويكردی تفسيري و منطقي مورد بررسی قرار گرفت. بر اساس نتایج، مفهوم شأن یک مفهوم پیچیده و چند بعدی است و عوامل متعددی با آن در ارتباط هستند و می‌توانند آن را ارتقاء داده یا تهدید نمایند. نتایج حاصل از بررسی و مقایسه مطالعات، موضوعات مفهوم شأن، ابعاد شأن، عوامل حفظ یا ارتقاءدهنده شأن و عوامل تهدیدکننده شأن در بیماران را پدیدار ساخت. نتیجه‌گیری: به‌کارگیری عوامل کلیدی شناسایی شده در پژوهش حاضر در زمینه عوامل مرتبط با شأن بیمار توسط پزشکان و پرستاران در مراقبت از بیماران مختلف در بالین و هم‌چنین هدایت پژوهش‌های بعدی در زمینه شأن بیمار می‌تواند سودمند باشد.
